# Tuberous sclerosis preclinical studies: timing of treatment, combination of a rapamycin analog (CCI-779) and interferon-gamma, and comparison of rapamycin to CCI-779

**DOI:** 10.1186/1471-2210-7-14

**Published:** 2007-11-06

**Authors:** Michael P Messina, Aubrey Rauktys, Laifong Lee, Sandra L Dabora

**Affiliations:** 1 Translational Medicine Division, Department of Medicine, Brigham & Women's Hospital, Karp Family Research Laboratories, Boston, MA, USA

## Abstract

**Background:**

Tuberous Sclerosis Complex (TSC) is an autosomal dominant hamartoma disorder with variable expression for which treatment options are limited. TSC is caused by a mutation in either the *TSC1 *or *TSC2 *genes, whose products, hamartin and tuberin, function as negative regulators in the highly-conserved mammalian target of rapamycin (mTOR) signaling pathway. Rapamycin (also known as sirolimus), an mTOR inhibitor, has been shown to reduce disease severity in rodent models of TSC and is currently being evaluated in clinical trials in human populations. The cytokine interferon-gamma (IFN-γ) is also a potential therapeutic agent for TSC. A high-expressing IFN-γ allele has been associated with reduced disease severity in human TSC patients and it has been shown in mouse models that treatment with exogenous IFN-γ reduces disease severity.

**Results:**

Here, we examine the effects of treating *Tsc2*^+/- ^mice at different time points with a rapamycin analog (CCI-779) as a single agent or with a combination of CCI-779 and IFN-γ. We observed that administering a short course of CCI-779 or CCI-779 plus IFN-γ reduced the severity of kidney lesions if administered after such lesions develop. As long as treatment is given after lesions arise, altering the time period during which treatment was given did not significantly impact the effect of the treatment on disease severity. We did not observe a significant benefit of combination therapy relative to treatment with a rapamycin analog alone in *Tsc2*^+/- ^mice. We also compared timing of treatment and two mTOR inhibitors (rapamycin and CCI-779) in nude mice bearing *Tsc2*^-/- ^tumors.

**Conclusion:**

Preventing the genesis of TSC-related kidney lesions in *Tsc2*^+/- ^mice is not an effective treatment strategy; rather, the presence of growing tumors appears to be the most important factor when determining an appropriate treatment schedule. Treatment with rapamycin was more effective in reducing tumor growth and improving survival in nude mice bearing *Tsc2*^-/- ^tumors and also resulted in higher rapamycin levels in blood, brain, and kidney tissue than treatment with an equal milligram dose of CCI-779. We anticipate these results will influence future preclinical and clinical trials for TSC.

## Background

Tuberous Sclerosis Complex (TSC) is an autosomal dominant tumor disorder that affects multiple organs, including the heart, lungs, brain, skin, and kidneys [1] and occurs at a frequency of about 1:6000 [2]. It has been estimated that 60–80% of TSC patients develop kidney angiomyolipomas (tumors composed of abnormal smooth muscle cells, fat cells, and blood vessels)[1]. A number of other medical issues, such as skin lesions (facial angiofibromas, shagreen patches, hypomelanotic macules, ungual fibromas, and forehead plaques), seizures, cognitive impairment, cortical tubers, cardiac rhabdomyomas, and in postpubertal females, TSC-related lymphangioleiomyomatosis (LAM), are also common in TSC patients[3].

TSC is caused by a loss of function of one of two genes, *TSC1 *or *TSC2*[2]. The products of these genes, hamartin and tuberin, function to negatively regulate mTOR in the highly conserved mTOR signaling pathway [4,5]. When tuberin and/or hamartin are absent or nonfunctional, mTOR is constitutively active and its downstream effectors, p70 S6 kinase (S6K), S6 ribosomal subunit (S6) and eukaryotic initiation factor 4E binding protein 1 (4EBP1) are hyperphosphorylated, which results in increased cell growth, cell proliferation, and survival[6,7]. Using compounds designed to inhibit mTOR is a common strategy in the investigation of possible treatments for TSC.

Rapamycin (Rapamune™ or sirolimus, Wyeth, Madison, NJ) is an FDA-approved mTOR inhibitor currently used to prevent rejection of solid organ transplants [8]. Rapamycin and its analogs have been successfully used to treat TSC-related lesions in rodent models [9-11] and rapamycin is currently being evaluated for its safety and efficacy in treating TSC-related lesions in human populations[12,13]. The mTOR pathway is also important in oncogenesis as PTEN, a tumor suppressor that functions upstream of mTOR, is mutated in many brain, prostate and other tumors[14]. Therefore, there is significant effort toward evaluating mTOR inhibitors as anti-cancer agents. There are currently four mTOR inhibitors (Sirolimus, CCI-779, RAD001, AP23575) being evaluated in a variety of malignancies including cancers of the brain, kidney, breast, ovaries, and lung as well as in leukemia and lymphoma [15]. CCI-779 (Torisel™ or temsirolimus, Wyeth) is now FDA-approved for the treatment of advanced renal cancer, and there is also some evidence for response to CCI-779 in glioblastomas, metastatic breast cancer, mantel cell non-Hodgkin's lymphoma, and Kaposi's sarcoma [16-20].

The cytokine interferon-gamma (IFN-γ) is another potential therapeutic agent for the treatment of TSC. It has been shown that the presence of a high-expressing IFN-γ allele significantly reduces the burden of kidney tumors in *Tsc2*^+/- ^mice relative to that of *Tsc2*^+/- ^mice with normal IFN-γ levels[21]. We have also observed an association between the presence of a high-expressing IFN-γ allele and reduced frequency of kidney angiomyolipomas in a cohort of human TSC patients [22].

Recently, we demonstrated that exogenous IFN-γ is an effective single agent in the treatment of TSC-related lesions in mouse models [10] and the combination of CCI-779 plus IFN-γ was more effective than single agents in a nude mouse model [11]. In our prior study, we showed that increased cell death along with decreased cell proliferation are important mechanisms underlying the antitumor activity of combination treatment in a nude mouse model for TSC-related tumors. Here, we have used *Tsc2*^+/- ^mice to investigate the effects of treatment with CCI-779 or a combination of CCI-779 plus IFN-γ at different time periods. We have also directly compared the efficacy of rapamycin with that of CCI-779 in a *Tsc2*^-/- ^tumor-bearing mouse model.

## Results

### Timing of Treatment and Combination Therapy in *Tsc2*^+/- ^Mice

**Figure 1:**
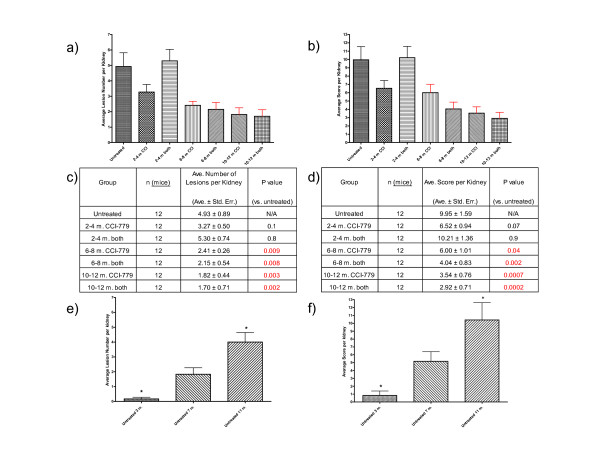
**Treatment with CCI-779 or CCI-779 plus IFN-γ reduced kidney tumor burden in *Tsc2*^+/- ^mice**. The cohorts treated with CCI-779 received 8 mg/kg by IP injection Monday through Friday for two months at the indicated ages. The cohorts treated with both agents received 8 mg/kg CCI-779 and 20,000 units IFN-γ by IP injection Monday through Friday for two months at the indicated ages. Severity of kidney disease was quantified both by counting the total number of cystadenomas and by scoring all the cystadenomas found. Average number of cystadenomas per kidney for indicated treatment cohort shown in bar graph (a) and table (c) format. Average cystadenoma score per kidney for indicated treatment cohort shown in bar graph (b) and table (d) format. Red error bars denote a statistically significant difference (P < 0.05) relative to untreated. "Both" in panels a-d refers to a combination of CCI-779 and IFN-γ. To illustrate the timing of kidney lesion genesis in untreated *Tsc2*^+/- ^mice, graphs of average number of cystadenomas per kidney (e) and average cystadenoma score per kidney (f) are shown for cohorts of mice at different ages (3, 7 and 11 months; n = 6 mice for each age cohort). The asterisks in panels e and f indicate that the severity of kidney disease in untreated cohorts at 3 months and 11 months differs significantly from disease severity at 7 months (t-test, P ≤ 0.05).

*Tsc2*^+/- ^mice were used for a seven-arm preclinical study to determine the impact of the timing of treatment for TSC renal disease and to compare treatment with CCI-779 to CCI-779 plus IFN-γ. The arms of the study are as listed in Table 1. All mice receiving drug treatment were treated for a two-month time period (2–4 months, 6–8 months, or 10–12 months). Because the primary goals were to evaluate timing of treatment with an mTOR inhibitor, and comparison of treatment with an mTOR inhibitor to the combination of an mTOR inhibitor plus IFN-γ, a group treated with single agent IFN-γ was not included in this experiment. The severity of kidney disease was evaluated using quantitative histopathology to obtain total lesion counts (Fig. 1a, c) and total kidney scores (Fig. 1b, d) as described in Methods. Because the development of kidney cystadenomas in *Tsc2*^+/- ^mice is age-dependent, disease severity was evaluated at age 52 weeks in all mice. To illustrate the timing of the genesis of kidney tumors in this mouse strain, the same methods were used to quantitate severity of kidney disease in six untreated mice (three males, three females) at each of three additional time points (3, 7 and 11 months, see Fig. 1e, f).

**Table 1:** Treatment cohorts of the *Tsc2*^+/- ^mouse experiment.

**Treatment**	**Number of Mice**	**Time Period of Treatment**	**Age at Euthanization**	**Average Weight at Necropsy (g) (Ave. ± Std. Error)**
Untreated	12 (6 M, 6 F)	N/A	12 months	34.0 ± 2.5
CCI-779	12 (6 M, 6 F)	2–4 months of age	12 months	33.4 ± 2.4
CCI-779 + IFN-γ	12 (6 M, 6 F)	2–4 months of age	12 months	32.1 ± 1.1
CCI-779	12 (6 M, 6 F)	6–8 months of age	12 months	31.7 ± 1.6
CCI-779 + IFN-γ	12 (6 M, 6 F)	6–8 months of age	12 months	29.2 ± 1.7
CCI-779	12 (6 M, 6 F)	10–12 months of age	12 months	30.5 ± 1.6
CCI-779 + IFN-γ	12 (6 M, 6 F)	10–12 months of age	12 months	28.6 ± 1.2

Compared with untreated controls, a significantly lowered burden of disease was observed, as evaluated both by tumor number and by tumor score, in all mice treated from 6–8 or 10–12 months of age, regardless of treatment (Fig. 1c, d). Treatment with CCI-779 or the combination of CCI-779 plus IFN-γ from 2–4 months was not effective. There was no significant difference between the 6–8 month cohort versus the 10–12 month cohort. Furthermore, no benefit was observed from adding IFN-γ to CCI-779 at any treatment time point. The lack of improvement with the combination therapy in this study using *Tsc2*^+/- ^mice differs from the results we have previously reported in nude mice bearing *Tsc2*^-/- ^tumors [11]. As shown (Fig. 1e, f), there are few kidney cystadenomas in untreated *Tsc2*^+/- ^mice at 3 months but they are easily observed at 7 months and the severity of kidney disease increases by 11 months.

### Subgroup analysis by kidney lesion type (cystic, papillary and solid) in treated and untreated *Tsc2*^+/- ^cohorts)

**Figure 2:**
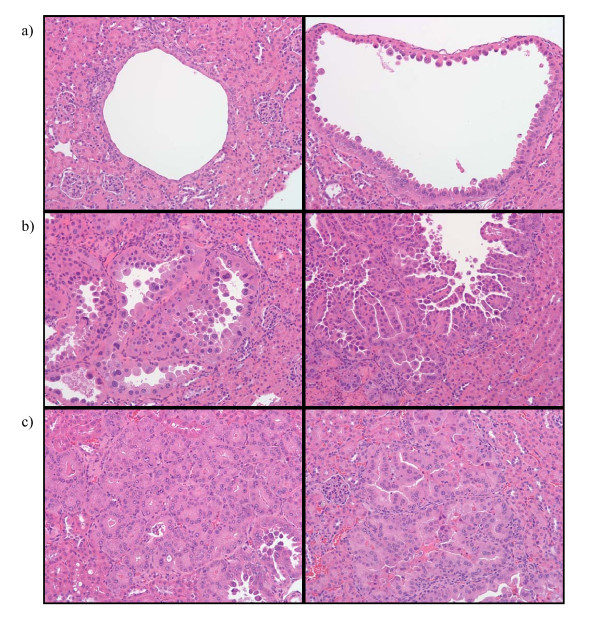
**Examples of the three different subgroups of kidney cystadenomas in *Tsc2*^+/- ^mice**. All pictures were taken at a 20× magnification. a) Cystic lesions. b) Papillary lesions. c) Solid lesions.

**Figure 3:**
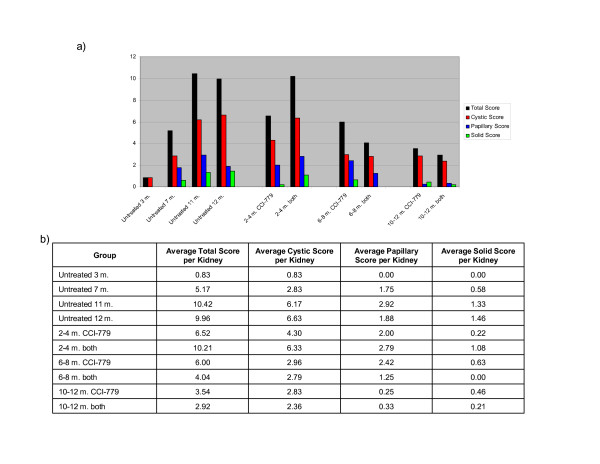
**Kidney cystadenoma scores according to treatment cohort and lesion subtype (cystic, papillary, solid)**. Cystadenoma score for each subtype is based on number and size of each lesion as described in methods. a) Average scores are plotted according to treatment cohort in graphical format. b) Average total score per kidney and average score per kidney for each lesion subtype by cohort in table format. "Both" refers to a combination of CCI-779 and IFN-γ.

Although we refer to all *Tsc2*^+/- ^mouse kidney lesions collectively as cystadenomas, they can be subdivided into 3 subtypes: cystic lesions, papillary lesions, and solid tumors (see Fig. 2). To investigate genesis of kidney cystadenomas in untreated *Tsc2*^+/- ^mice as well as the impact of treatment on cystadenoma subtype, kidney lesions were scored according to cystadenoma subtype (cystic, papillary, or solid). This subgroup data is shown for all treated and untreated *Tsc2*^+/- ^cohorts in Figure 3. Cystic lesions were observed to be the most common subtype in all cohorts. The untreated cohorts euthanized at different ages show that there tends to be an upward trend in all subtypes of kidney lesions between the ages of 3 to 12 months. Although treatment from 2–4 months was not significantly different than untreated controls, it is interesting to note that in the 2–4 month single agent CCI-779 cohort, there are fewer kidney lesions of all subtypes than the 2–4 month CCI-779 plus IFN-γ cohort. In the cohorts treated from 6–8 months, there are reduced numbers of cystic and solid lesions, but not of papillary lesions (when compared with 11 and 12 month untreated). When compared with the 7 month untreated cohort, there are similar numbers of cysts, papillary and solid lesions. In cohorts treated from 10–12 months, there are reduced numbers of cystic, papillary and solid lesions compared with the 11 and 12 month untreated cohorts. This data suggests that treatment with either CCI-779 alone or in combination with IFN-γ causes regression of all types of lesions (10–12 month treated vs. 11 and 12 month untreated). It therefore seems most likely that in the 6–8 month treated cohort, there is regression followed by regrowth of all lesion types.

### Timing of Treatment and Rapamycin vs. CCI-779 in a Nude Mouse Model of TSC

A nude mouse model of TSC was used to further investigate the impact of the timing of treatment and to compare rapamycin treatment to CCI-779. As described previously[10,11], nude mice were given subcutaneous injections of NTC/T2Null cells (*Tsc2*^-/- ^*Trp53*^-/-^, hereafter referred to as *Tsc2*^-/- ^cells) in the dorsal flank to induce development of TSC-related tumors. Mice were assigned to one of the following four treatment cohorts when their tumors reached the prescribed volume for their cohort: untreated (assigned when tumor volume was ~50 mm^3^), early rapamycin treatment (tumor volume ~50 mm^3^), late rapamycin (tumor volume ~250 mm^3^), and early CCI-779 (tumor volume ~50 mm^3^). Tumor volumes were measured and treatment was given daily Monday through Friday. All mice were euthanized when tumors exceeded 3000 mm^3^. To compare the cohorts, day 1 for mice in the early CCI-779 and early rapamycin treatment cohorts was taken to be the day the mouse received its first treatment and day 1 for mice in the untreated and late rapamycin treatment cohorts was taken to be the day on which that mouse had a tumor volume of approximately 50 mm^3^. Two methods were used to evaluate efficacy of drug treatment in the nude mouse model. Average tumor volumes were plotted for each cohort at all time points with 4 or more data points for treated cohorts and 3 or more data points for the untreated control cohort. The unpaired t test was used to compare tumor volumes from different cohorts on the last day on which there were 4 or more mice with tumor measurements (3 or more for the untreated control cohort because of the smaller sample size). Survival analysis was done by determining time to tumor size of 3000 mm^3 ^because animals with large tumors require euthanasia according to institutional animal care guidelines.

**Figure 4:**
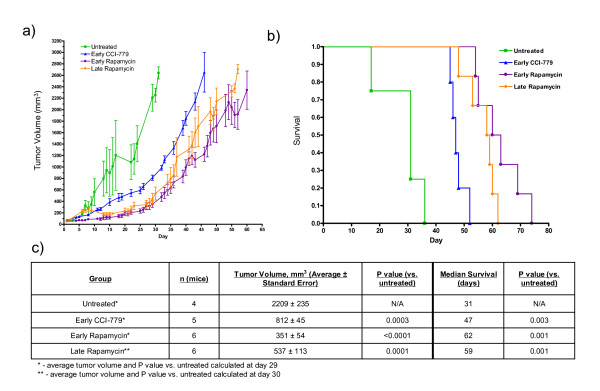
**Improved survival and decreased tumor growth in nude mice bearing *Tsc2*^-/- ^tumors due to treatment with either CCI-779 or rapamycin**. a) Average tumor growth over time for indicated treatment cohorts. b) Survival curve for indicated treatment cohorts. Mantel-Cox logrank analysis shows improved survival in all treatment cohorts relative to untreated. c) Data shown in table format. P values listed are in comparison to the untreated cohort.

As expected, all treatments significantly reduced tumor growth and improved survival (Fig. 4). At day 29, the average tumor volumes of the early CCI-779-treated cohort (812 ± 45 mm^3^) and the early rapamycin-treated cohort (351 ± 54 mm^3^) were lower than that of the untreated cohort (2209 ± 235 mm^3^). At day 30, the late rapamycin-treated cohort (537 ± 113 mm^3^) also had a lower tumor volume than the untreated cohort (2254 ± 120 mm^3^). Mantel-Cox logrank analysis shows improved survival in all three treatment cohorts when compared with untreated controls.

**Figure 5:**
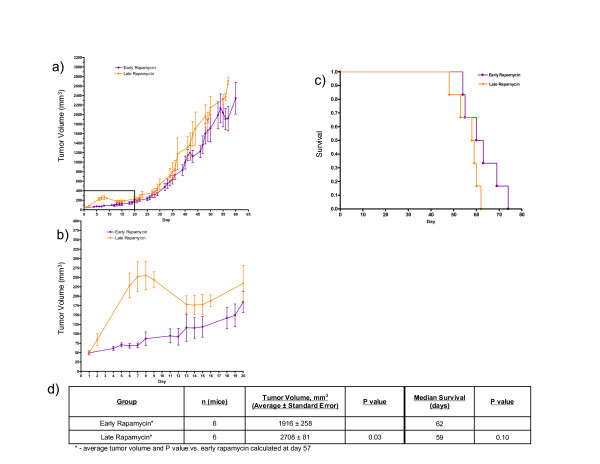
**Early rapamycin treatment compared to late rapamycin treatment in nude mice bearing *Tsc2*^-/- ^tumors**. a) Average tumor growth over time for the early rapamycin-treated and late rapamycin-treated cohorts only. b) Enlargement of the boxed portion of part a) illustrating the difference in growth pattern between the two treatment cohorts in the first 20 days of treatment. c) Survival curve for the early rapamycin and late rapamycin-treated cohorts. d) Data shown in table format.

To investigate treatment timing using this TSC mouse model, the early rapamycin cohort was compared to the late rapamycin cohort (Fig. 5). Although the early rapamycin cohort was observed to have a significantly lower tumor volume than the late rapamycin cohort on day 57 (1916 ± 258 mm^3 ^vs. 2708 ± 81 mm^3^, respectively; P = 0.03), there was no improvement in survival. The late rapamycin-treated cohort grew more sharply than the early rapamycin-treated cohort until approximately day 8, when the average tumor volume of the late rapamycin cohort reached 250 mm^3 ^and the mice in this cohort began to receive treatment; at this point, the average tumor volume of the late rapamycin cohort decreased until approximately day 15 when the average volume stabilized and the tumors entered a stage of progressive growth (Fig. 5b). From this point on, the growth curves of the two cohorts remained similar.

**Figure 6:**
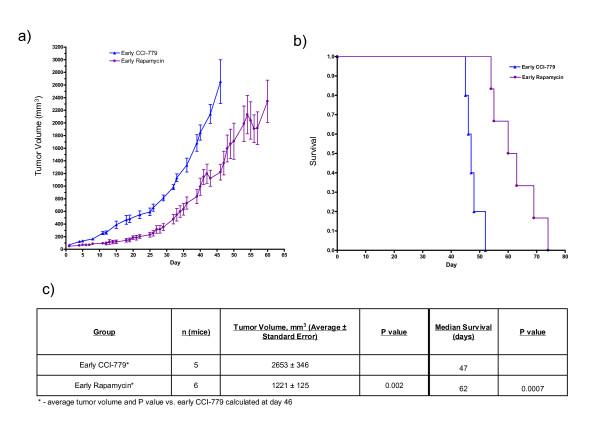
**Early rapamycin treatment is more effective than early CCI-779 treatment in nude mice bearing *Tsc2*^-/- ^tumors**. All animals were treated with 8 mg/kg of indicated drug five days per week. a) Average tumor growth over time for indicated cohorts. b) Survival curve for the early rapamycin and early CCI-779-treated cohorts. c) Data shown in table format.

CCI-779 is an injectable ester analog of rapamycin. It is more soluble than rapamycin and is known to be converted to rapamycin after injection[23]. Because we have used CCI-779 in our prior preclinical studies[10,11] and we are currently using rapamycin in an ongoing clinical trial[13], we were interested in comparing CCI-779 to rapamycin using our nude mouse model of TSC. As shown in Figure 6, early rapamycin treatment was compared with early CCI-779 treatment. Both drugs were given at 8 mg/kg five days per week (Monday-Friday). Although both drugs are effective when compared with untreated control (see Figure 4), rapamycin was more effective than CCI-779 in reducing tumor growth and improving survival. Tumor volume at day 46 was 2653 ± 346 mm^3 ^for the CCI-779 cohort and 1221 ± 125 mm^3 ^for the rapamycin cohort (P = 0.002, *t*-test). The median survival was 47 days for the CCI-779 cohort and 62 days for the rapamycin cohort (P = 0.0007, Mantel-Cox logrank analysis).

### Rapamycin levels in different tissues after treatment with rapamycin or CCI-779

**Figure 7:**
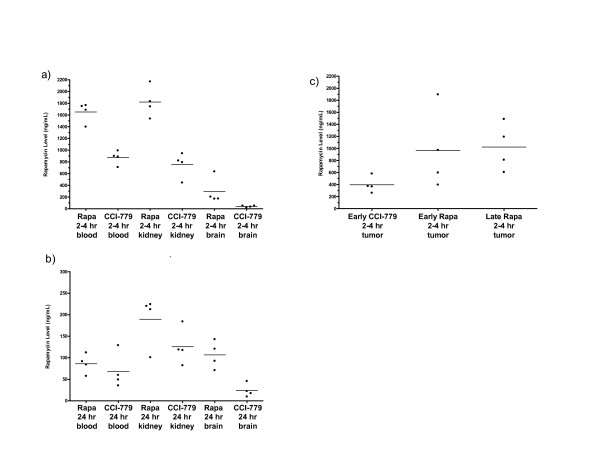
**Blood and tissue rapamycin levels from nude mice treated with rapamycin or CCI-779**. All mice were treated via IP injection at a dose of 8 mg/kg of indicated drug once per day and were euthanized either 2–4 hours or 24 hours after the final treatment was administered. Whole blood, brain, kidney, and tumor tissue were collected at necropsy. a) Blood, brain, and kidney rapamycin levels from cohorts treated with rapamycin or CCI-779 for four consecutive days and euthanized 2–4 hours after drug injection. b) Blood, brain, and kidney rapamycin levels from cohorts treated with rapamycin or CCI-779 for four consecutive days and euthanized 24 hours after drug injection. c) Tumor rapamycin levels from indicated cohorts treated with rapamycin or CCI-779 and euthanized 2–4 hours after drug injection. Rapa = rapamycin. This data is shown in table format with statistical analyses in Table 2.

Rapamycin levels were measured in cohorts of mice treated with either rapamycin or CCI-779 in order to investigate the tissue distribution of rapamycin and to compare levels after treatment with rapamycin vs. CCI-779. All mice in this experiment were given either rapamycin or CCI-779 at a dose of 8 mg/kg via intraperitoneal (IP) injection daily for four days to achieve steady state drug levels and to approximate the conditions of the nude mouse experiment. Rapamycin levels were measured in blood, brain, and kidney tissue from nude mice without tumors after either 2–4 hours or 24 hours after drug treatment (see Fig. 7a, b and Table 2a). These time points were selected based on pharmacokinetics of rapamycin and CCI-779 in humans and pilot studies of rapamycin blood levels in mice. In humans, rapamycin levels are known to peak 1–3 hours after oral dosing of rapamycin [24-26] and 0.74–2.26 hours after CCI-779 injection [16,27]. With rapamycin treatment in humans, there is an excellent correlation between 24 hour trough drug levels and area under the time-concentration curve (AUC) [26,28]. In pilot mouse studies of rapamycin level measurements done at 2–4 hours, 6 hours, 12 hours, 24 hours, and 48 hours after injection with rapamycin or CCI-779, measured rapamycin blood levels were highest at the 2–4 hour time point following treatment with either drug (data not shown).

**Table 2:** Average rapamycin levels (ng/mL) ± standard errors in blood, brain, kidney, and tumor tissue.

**Rapamycin levels at 2–4 hours and 24 hours in blood, kidney, and brain**						
	2–4 Hours Post-Treatment			24 Hours Post-Treatment		

	Rapamycin 8 mg/kg	CCI-779 8 mg/kg	P value*	Rapamycin 8 mg/kg	CCI-779 8 mg/kg	P value*

Number of Mice	4	4		4	4	
Whole Blood	1650 ± 1715	872 ± 119	0.0003	86 ± 23	68 ± 42	NS
Brain Homogenates	295 ± 227	39 ± 14	NS (0.06)	107 ± 32	24 ± 16	0.003
Kidney Homogenates	1820 ± 264	751 ± 215	0.0003	190 ± 59	126 ± 43	NS

**Rapamycin levels at 2–4 hours in tumors from the nude mouse experiment**						

	2–4 Hours Post-Treatment					

	Early CCI-779	Early Rapamycin	Late Rapamycin	P value**		

Number of Mice	4	4	4			
Tumor Homogenates	395 ± 134	965 ± 665	1024 ± 393	NS		

*Drug levels from rapamycin cohort compared with CCI-779 cohort for indicated tissue and time point, NS = not significant.

**Tumor drug levels from early CCI-779 cohort and late rapamycin cohort were compared with early rapamycin cohort, NS = not significant

Rapamycin levels were also measured in four tumors from each cohort from the nude mouse experiment (all tumor tissue was obtained 2–4 hours after the final drug treatment). The results are shown in Fig. 7c and Table 2b. At 2–4 hours after drug treatment, the average rapamycin concentration was similar in blood and kidney tissue, but lower in brain tissue. At 2–4 hours, rapamycin levels were ~2 times higher in blood and kidneys from the rapamycin-treated animals compared with the CCI-779-treated animals (P < 0.05). Interestingly, at 2–4 hours, rapamycin levels were 7–8 times higher in brain tissue from the rapamycin-treated animals compared with the CCI-779-treated animals (this difference approached statistical significance, P = 0.06). At 2–4 hours, blood rapamycin levels were 1.6–2.2 times higher in blood compared with tumor tissue after treatment with either rapamycin or CCI-779.

At 24 hours after rapamycin treatment, rapamycin concentration was highest in kidney tissue and similar in blood and brain tissue with brain > blood. At 24 hours after CCI-779 treatment, rapamycin levels were higher in kidney tissue than in brain and blood with blood > brain. At 24 hours, rapamycin levels were 1.3–1.5 times higher in blood and kidneys from the rapamycin-treated animals compared with the CCI-779-treated animals. Interestingly, at 24 hours, rapamycin levels were 4–5 times higher in brain tissue from the rapamycin-treated animals compared with the CCI-779-treated animals (P = 0.003).

These results show that average rapamycin levels in all tissues are higher after rapamycin treatment compared with CCI-779 treatment at both 2–4 hours and 24 hours. Because CCI-779 is converted to rapamycin, it is not surprising to see that the tissue levels of rapamycin differ significantly at the early time point. It is interesting to see that comparison of rapamycin treated and CCI-779 treated animals at 24 hours shows that differences in rapamycin levels are not significant in blood and kidney tissue. Despite the similarity in blood and kidney levels, the brain rapamycin concentrations are significantly higher after rapamycin injection.

## Discussion

The *Tsc2*^+/- ^mouse is a useful model for the kidney angiomyolipomas that develop in TSC. Both the renal cystadenomas that occur in *Tsc2*^+/- ^mice and kidney angiomyolipomas that occur in individuals with TSC tend not to be present at young ages but develop over time. In our preclinical study using *Tsc2*^+/- ^mice, we sought to evaluate the importance of timing of a two-month treatment period with either CCI-779 or CCI-779 plus IFN-γ. Our results show that if treatment is initiated prior to the appearance of the kidney lesions (at 2–4 months), the treatment was not effective. Once kidney lesions are present, a two-month course of treatment does reduce kidney disease severity, but there was no difference between treating at 6–8 months versus 10–12 months. Our data suggests that there is cystadenoma regression then regrowth in the cohort treated at 6–8 months, raising the possibility that there may be benefit to longer term treatment or continuing a lower maintenance dose to reduce regrowth of kidney lesions.

There are three studies showing that IFN-γ as a single agent may be a useful therapeutic agent for TSC kidney disease [10,21,22]. In a mouse genetic study, the frequency of kidney tumors was dramatically reduced in *Tsc2*^+/- ^mice engineered to express high levels of endogenous IFN-γ [21]. In a genetic association study, we demonstrated that a high expressing allele for IFN-γ is associated with a lower frequency of kidney angiomyolipomas in patients with known TSC2 mutations [22]. These genetic data in mice and humans suggest that long term exposure to high levels of IFN-γ may be required for IFN-γ to reduce the severity of TSC related kidney disease. In our prior preclinical study using *Tsc2*^+/- ^mice [10], single agent IFN-γ was administered for 10 months (from age 2 months-12 months) and there was significant reduction in the severity of kidney disease. We have previously compared the combination of CCI-779 plus IFN-γ with single agent CCI-779 and single agent IFN-γ in nude mice bearing *Tsc2*^-/- ^tumors and found a significant reduction in tumor growth and improved survival with combination therapy [11]. In contrast, here we evaluated combination CCI-779 plus IFN-γ using *Tsc2*^+/- ^mice and found no benefit to adding the IFN-γ to CCI-779 in this study. After considering the details of these relevant prior studies, we conclude that this discrepancy is likely due to the shorter IFN-γ treatment duration (2 months) compared with our prior study (10 months) using *Tsc2*^+/- ^mice, along with insufficient power to detect a minor difference between the groups. A comparison of our prior studies using nude mice bearing TSC-related tumors also suggests that early treatment with IFN-γ yields a better response. In Lee et al., 2005 we treated animals on day 18 after *Tsc2*^-/- ^cells were injected and tumors were small (range 0–32 mm^3^, average of 6.7 mm^3^) and we observed a dramatic improvement with IFN-γ treatment. In Lee et al., 2006, the study design differed as IFN-γ treatment was initiated when subcutaneous tumors were significantly larger (500 mm^3^), and although single agent IFN-γ was still somewhat effective, the response was subtle. In the *Tsc2*^+/- ^study reported here, we observed that the combination treatment cohort had a lower cystadenoma score than the single-agent CCI-779 cohort in both the 6–8 month and 10–12 month groups, although this difference was not statistically significant (Figure 1a–d). It is possible that early and prolonged IFN-γ treatment along with larger cohorts might allow us to observe a difference between combination treatment versus single-agent CCI-779 treatment in *Tsc2*^+/- ^mice, and these issues will be considered in the design of future preclinical studies. It was surprising to see that when we compare the group treated with single agent CCI-779 from 2–4 months to the group treated with CCI-779 plus IFN-γ from 2–4 months, single agent CCI-779 looks significantly better than combination treatment (P = 0.03). This finding is puzzling as it is not consistent with other treatment time points in this study or findings in our prior studies on combination therapy, and we do not have a good explanation for this difference. Since neither 2–4 month treatment group differed significantly from the untreated control group, we conclude that the difference between CCI-779 and combination treatment at this time is not important.

Loss of heterozygosity has been observed in kidney angiomyolipomas[29] and subependymal giant cell tumors[30]. Because TSC is a tumor suppressor gene syndrome, nude mice bearing *Tsc2*^-/- ^tumors are a useful generic model for TSC-related tumors. We used this model to investigate treatment timing and to compare two mTOR inhibitors. In our comparison of treatment initiated at tumor size of 50 mm^3 ^vs. tumor size of 250 mm^3^, we found that there was a statistically significant reduction in tumor volume with earlier rapamycin treatment but no survival advantage. In the later rapamycin treatment cohort, the tumors underwent regression then regrowth. This is evidence that there is response followed by development of resistance. We have shown previously that progressive tumor growth occurs even though the mTOR pathway is inhibited (as demonstrated by reduced pS6) [11]. Although dramatic benefit of earlier treatment was not observed in this experiment, there may be a slight advantage of earlier treatment as we did observe a reduction of tumor volume in the early treatment cohort.

Because our prior preclinical studies have used the rapamycin analog, CCI-779, and rapamycin is being used in ongoing clinical trials, we sought to demonstrate that rapamycin and CCI-779 were equally effective using our nude mouse model for TSC. To our surprise, we found that although both drugs were effective, rapamycin was more effective than CCI-779 when given at the identical dose (in milligrams) as demonstrated by reduced tumor growth and improved survival. Since CCI-779 is an ester analog of rapamycin that is known to be a prodrug which is converted to rapamycin after injection[23], we evaluated rapamycin levels in blood, brain, tumor and kidneys after injection with either rapamycin or CCI-779. We found that average rapamycin levels are higher in blood, kidneys, brain, and tumor tissue 2–4 hours and 24 hours after rapamycin treatment compared with CCI-779 treatment. At 24 hours, the difference in rapamycin levels from the two treatment groups was statistically significant only in brain tissue and not in blood or kidney tissue. Although a more detailed analysis with additional time points and larger numbers of animals is required to understand the pharmacokinetic and phamacodynamic properties of rapamycin versus CCI-779 in nude mice, our observation that average rapamycin levels are higher after rapamycin treatment at both 2–4 hours and 24 hours in all tissues is consistent with our finding that rapamycin is more effective than CCI-779, as measured by tumor growth and survival analysis in nude mice bearing TSC-related tumors. These results coupled with the fact that rapamycin has been approved for human use for many years and consequently has a well-known toxicity profile make rapamycin our first choice of mTOR inhibitors for future TSC clinical trials. If neurologic toxicity is observed with rapamycin in human TSC studies, our results suggest that CCI-779 may be a useful alternative.

## Conclusion

In both the *Tsc2*^+/- ^mouse model and nude mouse model for TSC tumors, the timing of initiation of mTOR inhibitor treatment of TSC-related tumors does not appear to be important, provided that tumors are actively growing at the time treatment is initiated. Attempting to prevent the genesis of kidney lesions in *Tsc2*^+/- ^mice using short term mTOR inhibitor treatment is not an effective strategy. Treatment with a combination of IFN-γ and an mTOR inhibitor for two months did not prove to be more effective than an mTOR inhibitor alone in *Tsc2*^+/- ^mice. This result differs from our findings in the nude mouse tumor model [11] and could be due to the shorter duration of IFN-γ therapy used here in *Tsc2*^+/- ^mice. Finally, rapamycin proved to be more effective than its analog CCI-779 at equal doses. Rapamycin treatment results in higher brain, kidney and tumor levels of rapamycin than treatment with an equal dose of CCI-779. As TSC is a multi-system disorder that affects the brain, kidneys and other organs, depending on TSC disease manifestations and toxicity profile, it may ultimately be advantageous to have mTOR inhibitors with differing tissue distribution profiles. We anticipate these preclinical studies will influence the design of future preclinical studies and clinical trials for TSC.

## Methods

### *Tsc2*^+/- ^mice and treatment with CCI-779 or CCI-779 plus IFN-γ

The *Tsc2*^+/- ^mice are heterozygous for a deletion of exons 1–2 and have been described previously[31]. The *Tsc2*^+/- ^cohort used in these experiments was generated from a cross with wild-type C57BL/6 mice. Sibling littermates were used as controls to avoid bias due to strain variation. *Tsc2*^+/- ^mice were assigned to one of seven cohorts based on treatment given and the time period in which treatment was given. All treatments were given by IP injection. Cohorts included treatment with either CCI-779 alone or CCI-779 in combination with IFN-γ, and treatment was given from 2–4 months of age, 6–8 months of age, or 10–12 months of age (Table 1). All mice in these cohorts were euthanized at 52 weeks of age according to institutional animal care guidelines. Mice treated from 10–12 months of age were euthanized within 2–4 hours after the final treatment was given. The severity of kidney disease was determined in all animals using quantitative histopathology as described below.

*Tsc2*^+/- ^mice were treated for 2 months with 8 mg/kg CCI-779 or a combination of 8 mg/kg CCI-779 and 20,000 units murine IFN-γ administered by IP injection daily Monday through Friday. This dose and schedule were selected on the basis of earlier experiments [11]. CCI-779 powder was obtained from Wyeth (Madison, NJ). A 30 mg/mL stock of CCI-779 was made in ethanol (stored at 20°C for up to one week), diluted to 1.2 mg/mL in vehicle (5% PEG, 5% Tween-80) and administered within 24 hours. Murine IFN-γ (R&D Systems, Minneapolis, MN) was diluted to 100,000 units/mL in PBS containing 0.1% mouse serum albumin (Sigma-Aldrich, Inc., St. Louis, MO), stored at 4°C, and administered within 24 hours. All animals were checked 5 times per week and their general behavior was monitored. Animals were weighed weekly, and at the time of necropsy, there were no significant differences in weight between cohorts (average weights at necropsy shown in Table 1). All experiments were done according to animal protocols approved by our institutional animal protocol review committee (Children's Hospital Boston, Boston, MA) and were compliant with federal, local, and institutional guidelines on the care of experimental animals.

### Quantitation of kidney cystadenomas in *Tsc2*^+/- ^mice by histopathology

For quantitative histopathologic examination, each kidney was fixed and sliced at 1 mm intervals. The slices were then arranged sequentially for paraffin embedding, sectioning, and staining with hematoxylin and eosin (H&E). Slides were coded and all cystadenomas were counted, measured, and scored according to the scale shown in Table 3 by a blinded observer (AR). For cystadenomas that extended into more than one 1 mm kidney slice, only the largest cross-sectional area was scored and used for the analyses. Lesions smaller than 0.01 mm^2 ^in cross-sectional area were not counted towards the analyses. To illustrate the timing of kidney tumor genesis in untreated *Tsc2*^+/- ^mice from this colony, kidneys were also collected from untreated mice at 3, 7 and 11 months.

**Table 3:** Scoring scale for kidney cystadenomas.

**Score**	**Area Range (mm^2^)**
1	0.01 < x ≤ 0.09
2	0.09 < x ≤ 0.2
3	0.2 < x ≤ 0.35
4	0.35 < x ≤ 0.5
5	x > 0.5

Areas of cystadenomas were calculated by multiplying the longest axis and its perpendicular axis.

Because the kidney cystadenomas that occur in *Tsc2*^+/- ^mice can be divided into subgroups that include cystic, papillary and solid lesions, we use the term "kidney cystadenomas" to refer to the entire spectrum of kidney lesions observed. In addition to analyzing data according to all cystadenomas, a subgroup analysis was also done by coding cystic, papillary, and solid kidney lesions separately. For the subgroup analysis, lesions with areas that were ≤25% filled with abnormal cells were labeled as cystic, lesions with areas that were >25% filled in with abnormal cells were labeled as papillary, and those that were completely filled with abnormal cells were labeled as solid. Examples of each of these types of lesions can be found in Figure 2. Subgroup data is presented as kidney lesion score per kidney where the subgroup score is based on the number and size of each type of lesion as described above.

### Induction of subcutaneous *Tsc2*^-/- ^tumors in nude mice and treatment with CCI-779 or rapamycin

Nude mice (strain CD-1nuBR, 7 weeks old) were obtained from Charles River Laboratories (Wilmington, Massachusetts). 24 nude mice were injected subcutaneously on the dorsal flank with 2.5 million NTC/T2null (*Tsc2*^-/-^*, Trp53*^-/-^) cells, as in previous studies[10,11]. Tumors were measured with calipers daily Monday through Friday. Tumor volumes were calculated by use of the formula *L *× *W *× *W *× 0.5[32]. Mice were randomly assigned to four cohorts of six mice each at the time small tumors were visible with volumes ~5–10 mm^3^. Cohorts included untreated, early CCI-779-treated, early rapamycin-treated, and late rapamycin-treated. Mice in the early CCI-779- and early rapamycin-treated cohorts began treatment when tumors reached a volume of approximately 50 mm^3 ^and mice in the late rapamycin treated cohort began treatment when tumors reached a volume of approximately 250 mm^3^. All treatments were given daily Monday through Friday via IP injection. Mice were euthanized when tumor volumes exceeded 3000 mm^3^, and within 2–4 hours after the final treatment. All tumor-bearing animals were euthanized according to institutional animal care guidelines based on tumor size or presence of an open ulcer. Tumor tissue was harvested for rapamycin level testing as described below.

We excluded three mice from our analysis of this experiment. One mouse assigned to the early CCI-779 cohort was euthanized with a small tumor (<50 mm^3^) prior to CCI-779 treatment because of weight loss and dehydration that appeared to be unrelated to drug treatment or tumor burden. Two mice assigned to the untreated cohort were housed with mice undergoing treatment with high doses of topical CCI-779 or rapamycin ointment as part of a separate pilot experiment. These animals were excluded from the data analysis because it was determined that this environmental exposure to rapamycin or CCI-779 affected tumor growth.

Treatment with CCI-779 for the nude mouse experiment was prepared and administered as described above. Rapamycin powder was obtained from LC Laboratories (Woburn, MA) and a 20 mg/mL stock was made in ethanol (stored at 20°C for up to one week), diluted to 1.2 mg/mL in vehicle (5% PEG, 5% Tween-80) and administered within 24 hours. All animals were checked 5 times per week and their general behavior was monitored. Animals were weighed weekly, and at the time of necropsy, there were no significant differences in weight between cohorts (data not shown). We did not observe significant toxicity from treatment with rapamycin or CCI-779 at the doses used in this study.

The study design of this experiment differs from our prior study designs [10,11]. In [10], all treatment started on the same day regardless of tumor size and in [11], treatments were started when tumor volumes were 500 mm^3^. Methods for determining rapamycin levels in tumors from this experiment are described below.

### Rapamycin levels in tumors and other tissues

Rapamycin levels were measured from *Tsc2*^-/- ^tumor samples from all treatment cohorts in the nude mouse experiment described above. Tumors were harvested 2–4 hours after the final treatment and then 200 mg of tumor tissue was homogenized in 1 mL of sterile saline. Rapamycin levels were measured by the Clinical Laboratory at Children's Hospital Boston (Boston, MA). To further investigate the tissue distribution of rapamycin after treatment with either rapamycin or CCI-779, rapamycin levels were also measured in blood, kidneys and brains from nude mice with no tumors. For these measurements, sixteen nude mice of the same strain and age used in the nude mouse tumor experiment described above were treated with an 8 mg/kg dose of either rapamycin or CCI-779 daily for four days. Blood and tissues were obtained either 2–4 hours or 24 hours after the final dose (see Table 2). Whole blood was drawn into a syringe via cardiac puncture, dispensed into an EDTA-containing blood tube, and diluted with an equal volume of sterile saline to ensure sufficient volume for rapamycin level analysis. Brains and kidneys were snap-frozen in liquid nitrogen upon collection and were later thawed and homogenized in sterile saline at a concentration of 200 mg of tissue per mL of saline. Rapamycin levels were measured by the Clinical Laboratory at Children's Hospital Boston (Boston, MA). All measured rapamycin levels were then corrected according to sample dilution.

### Statistical Analyses

GraphPad Prism software (version 4.01) was used for all statistical analyses, and *P *≤ 0.05 was considered to indicate significance. All results were replicated independently from raw data by two observers (AR and MM). The *t *test was used for quantitative analyses and Mantel-Cox logrank analysis was used for survival data where the time of death is the time of euthanasia due to tumor size of 3000 mm^3 ^or larger.

## Authors' contributions

SD provided funding, critical guidance for the experiments, and was responsible for supervising the writing and editing of the manuscript.

LL assisted in planning and performing the experiments as well as assisted in critical evaluation and editing of the manuscript.

AR performed the histopathological analysis for the *Tsc2*^+/- ^mouse experiment as well as assisted in performing the nude mouse experiment. Along with MM, AR performed the statistical analysis for both experiments.

MM performed the experiments, performed the statistical analyses for both experiments with AR, and drafted and edited the manuscript.

All authors have read and approved the final manuscript.
